# Identification of a novel heterozygous germline *RAD52* missense mutation in a patient with gallbladder carcinoma: A case report

**DOI:** 10.1097/MD.0000000000025957

**Published:** 2021-05-14

**Authors:** Wenhu Zhao, Yongjiu Dai, Lei Yue, Jian Gu, Erhong Meng, Dongliang Wang, Siyao Liu, Xinyin Han, Xintong Wang, Guojun Li, Xinzheng Dai

**Affiliations:** a Hepatobiliary Center, the First Affiliated Hospital of Nanjing Medical University, Nanjing; b ChosenMed Technology (Beijing) Co. Ltd; c Computer Network Information Center, Chinese Academy of Sciences; d University of the Chinese Academy of Sciences, Beijing, China.

**Keywords:** case report, family pedigree, gallbladder carcinoma, germline mutation, RAD52

## Abstract

**Rationale:**

Gallbladder carcinoma is a malignant biliary tract tumor which is characterized by poor prognosis. Recent advances in genomic medicine have identified a few novel germline mutations that contribute to the increased risk of gallbladder carcinoma. *RAD52* is a crucial human deoxyribonucleic acid (DNA) repair gene involved in maintaining genomic stability and preventing tumor occurrence.

**Patient concerns:**

A 57-year-old man was hospitalized for space-occupying lesions in the gallbladder.

**Diagnosis:**

A diagnosis of gallbladder adenocarcinoma was made based on computed tomography, B-ultrasound, blood tests, and postoperative pathology.

**Interventions:**

Next-generation sequencing using a 599-gene panel and Sanger sequencing were performed to validate the mutation in the proband and his family members, respectively.

**Outcomes:**

A novel potentially pathogenic heterozygous germline *RAD52* missense mutation (c.276T > A: p.N92K) was identified in the patient. Sanger sequencing revealed that this variation was not observed in unaffected family members.

**Lessons:**

We identified a novel heterozygous germline *RAD52* missense mutation in a patient with gallbladder carcinoma. Our results added to the current body of knowledge. It also provides new insights into genetic counseling and targeted therapeutic strategies for patients with gallbladder carcinoma.

## 1 Introduction

Gallbladder carcinoma (GBC) is a biliary tract carcinoma (BTC) with a poor prognosis. Although the significant risk factors for GBC include gallstones, chronic inflammation, and gallbladder polyps, inherited and other predisposing factors might also contribute to the carcinogenesis of GBC.^[1]^ Wardell et al reported that a few deleterious germline mutations in genes, such as *BRCA1/2*, *RAD51D*, *MLH1*, and *MSH2*, were identified in approximately 11% of patients with BTC.^[2]^

Genome integrity is a characteristic of all organisms and its maintenance is critical for disease prevention. The genome is exposed to a variety of genotoxic agents that can lead to deoxyribonucleic acid (DNA) damage, including the most harmful type of damage, double-stranded breaks (DSBs).^[3]^ For this reason, there are pathways to repair DSBs to maintain the stability of the genome and prevent diseases. Previous studies have reported that *RAD52* plays a vital role in different DSB repair pathways.^[4]^ Notably, *RAD52* variants have been associated with increased risk of various cancers such as lung cancer,^[5–7]^ glioma,^[8]^ breast cancer,^[9]^ hepatocellular carcinoma,^[10,11]^ and colorectal cancer.^[12]^ However, to the best of our knowledge, no association has been reported between *RAD52* and GBC.

Here, we detected a novel heterozygous germline missense mutation in *RAD52* in a patient with GBC. Variations were not detected in unaffected family members.

## 2 Ethics and methods

This study was approved by the Ethics Committee of the First Affiliated Hospital of Nanjing Medical University. Written informed consent was obtained from the individuals for participation and for the publication of potentially identifiable images or data included in this study.

Genomic DNA from the peripheral blood mononuclear cell fractions was extracted using the MagPure Tissue & Blood DNA Kit (Cat#D6315; Beijing ComWin Biotech Co., Ltd, Beijing, China) according to the manufacturer's instructions. After quality control of the extracted DNA samples, a library was prepared according to a 599-gene next-generation sequencing panel in a laboratory accredited by the College of American Pathologists (CAP) [ChosenONE 599, ChosenMed (Beijing) Technology Co. Ltd, Beijing, China]. Sanger Sequencing was conducted according to literature (Tsingke Biotechnology Co., Ltd. Beijing, China).^[13]^ Three bioinformatics programs including MutationTaster, SIFT (Sorting Intolerant From Tolerant) and PolyPhen-2 (Polymorphism Phenotyping v2) were used to predicted the effect of novel mutations on protein function.

## 3 Case presentation

A 57-year-old man presented to our hospital with space-occupying lesions in the gallbladder. Computed tomography revealed a blurred and unevenly dense gallbladder. B-ultrasound showed several hypoechoic areas approximately 33 × 31 mm in size in the left lobe of the liver with unclear boundaries and without high blood flow. An irregular echo was detected in the gallbladder, 69 × 39 mm in size, with a hypoechoic margin but a hyperechoic center. An enhanced computed tomography scan revealed an irregular and thickened gallbladder with accumulation of fluid.

The blood test results showed alpha-fetoprotein 2.12 ng/mL, carcinoembryonic antigen 9.93 ng/mL (high), carbohydrate antigen 19-9 34.44 ng/mL, and a liver function test revealed total bilirubin 4.5 umol/L, total protein 57.2 g/L, albumin 38.3 g/L, and globulin 18.9 g/L.

**Figure 1:**
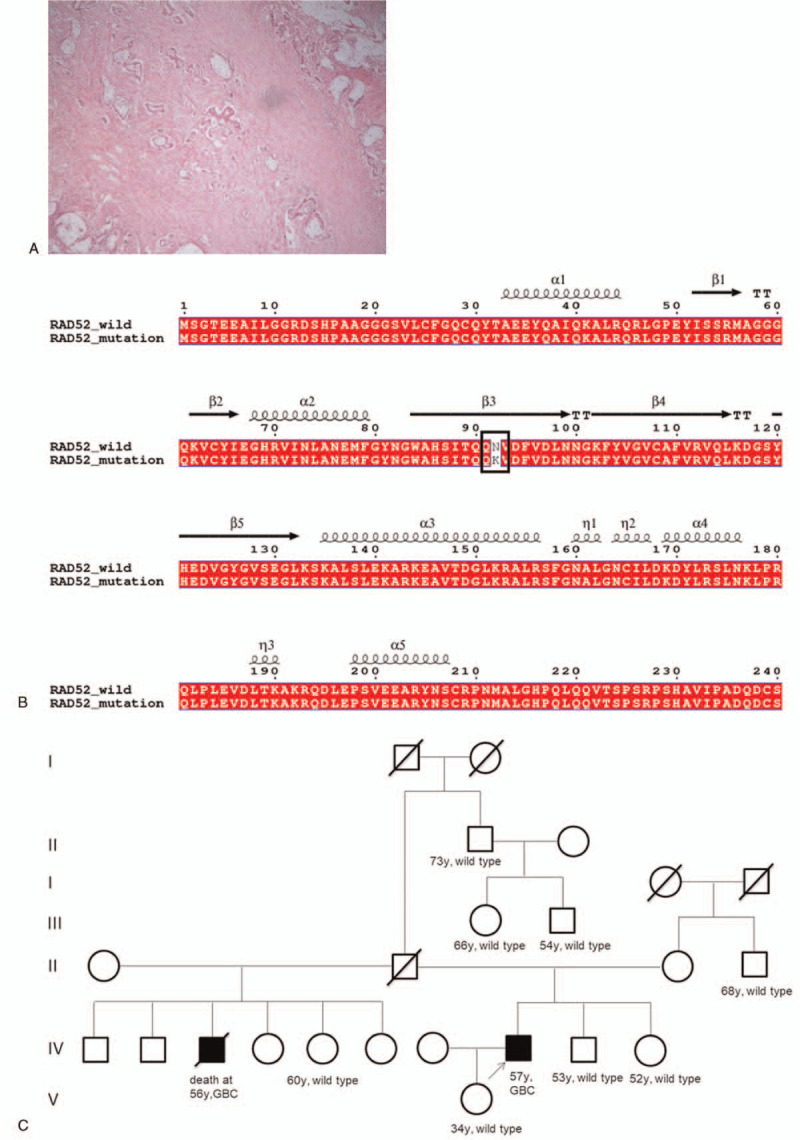
Pathological examination, sequence alignment of wild-type and mutant RAD52 protein, and a pedigree chart. A) Pathological examination of gallbladder cancer tissues. Hematoxylin eosin staining (400×). B) Sequence alignment of wild-type and mutant RAD52 protein. C) Pedigree chart. The status of each person was indicated as wild-type or as harboring the N92K mutation (N92K). The proband is indicated by an arrow.

The patient underwent surgical treatment. Intraoperative exploration revealed a hard gallbladder measuring 7 × 6 × 3 cm in size. The postoperative pathology confirmed gallbladder adenocarcinoma (Fig. 1A) which had spread to the liver and lymph nodes. There was no follow-up information after the patient was discharged.

The 599-gene next-generation sequencing blood panel revealed a novel heterozygous germline *RAD52* missense mutation (NM_134424: exon4: c.276T > A: p.N92K). This variation has never been reported in any database or any publications, such as the Exome Aggregation Consortium and 1000 Genomes Project. The *RAD52* mutation frequency was 41.27%. Three bioinformatics software tools (MutationTaster, SIFT, and PolyPhen-2) consistently predicted that this mutation could affect the function of the *RAD52* protein and was pathogenic. The sequence alignment results of wild-type and mutant *RAD52* proteins are shown in Figure 1B. It is noteworthy that the results predicted by these 3 bioinformatics tools were not concordant for the remaining germline variants of uncertain significance, except RAD52 (Table 1).

**Table 1:** Characteristics of germline variants of uncertain significance and analysis of predicted protein structure and disease-causing effects in the proband.

Gene	Transcript	Chromosome	Exon	Nucleotide Change	AAChange	CLinVar	ExAC/1000G	Novel	MutationTaster	SIFT	Polyphen-2
PRKD1	NM_002742	chr14	15	c.2084G > A	p.R695Q	No report	Known variant	Not novel	Disease causing	Tolerated	Possibly damaging
LRP1B	NM_018557	chr2	4	c.401A > G	p.N134S	No report	Unknown variant	Novel	Disease causing	Tolerated	Benign
EZH1	NM_001991	chr17	4	c.202G > C	p.V68L	No report	Known variant	Not novel	Disease causing	Tolerated	Benign
KMT2A	NM_001197104	chr11	7	c.3638T > C	p.V1213A	No report	Unknown variant	Novel	Disease causing	Tolerated	Benign
RAD52	NM_134424	chr12	4	c.276T > A	p.N92K	No report	Unknown variant	Novel	Disease causing	Damaging	Probably damaging
CUL4A	NM_001008895	chr13	6	c.577A > G	p.I193V	No report	Unknown variant	Novel	Disease causing	Tolerated	Benign
IGF2	NM_000612	chr11	4	c.512C > G	p.A171G	No report	Known variant	Not novel	Polymorphism	Damaging	Probably damaging
EPHA5	NM_001281766	chr4	5	c.1288G > A	p.D430N	No report	Known variant	Not novel	Disease causing	Tolerated	Probably damaging
AXIN1	NM_003502	chr16	6	c.1750G > A	p.A584T	No report	Known variant	Not novel	Polymorphism	Tolerated	Benign

**Figure 2:**
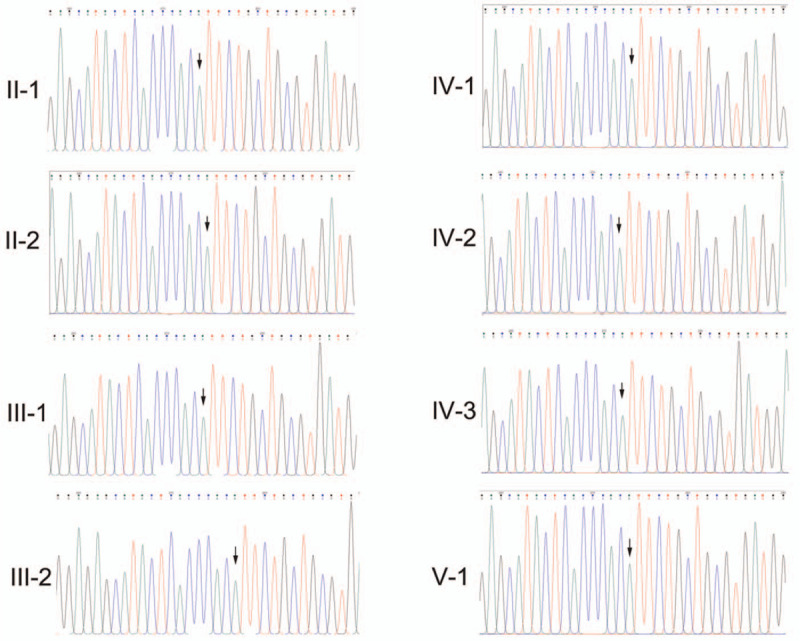
Absence of the germline N92K mutation in the unaffected family members as determined by Sanger sequencing.

Sanger sequencing demonstrated that the same variation was not detected in the unaffected family members (Fig. 2).

According to the ACMG/AMP guidelines in 2015,^[14]^ the *RAD52* missense variant seems to be pathogenic moderate 2 (PM2) and pathogenic supporting 3 (PP3), with a possibility of pathogenic supporting 1(PP1).

## 4 Discussion

GBC is the most common type of cancer in the biliary tract. Notably, hereditary susceptibility is an important risk factor that plays a role in the development of GBC. Previous studies on understanding germline mutations underlying tumorigenesis have provided novel insights into the development of GBC.

Studies have demonstrated that DNA repair plays a vital role in genomic maintenance to prevent carcinogenesis. Furthermore, RAD52 participates in a variety of DNA repair pathways activated by DSBs, such as homologous recombination which is one of the major repair pathways, single-strand annealing, break-induced replication, and microhomology-mediated end-joining.^[4,15,16]^*RAD52* plays an important role by stimulating complementary single-stranded DNA annealing and the activity of *RAD51* recombinase.^[15]^ In addition, investigations have demonstrated that *RAD52* might have a synthetic lethality relationship with PALB2 or BRCA2 in the process of maintaining genome integrity and preventing cancer development.^[8]^

To date, there have been no reports of *RAD52* germline mutations in patients with GBC. Since both *RAD51* and *RAD52* belong to homologous recombination genes,^[17]^ and germline *RAD51D* has been detected in approximately 11% of patients with BTC,^[2]^ we hypothesized that *RAD52* might also contribute to the pathogenesis of GBC. In this study, we detected a novel heterozygous germline *RAD52* missense mutation (NM_134424: exon4: c.276T > A: p.N92K) in a 57-year-old male patient with GBC, which was predicted to be consistently pathogenic by 3 bioinformatics software tools. Sanger sequencing revealed that the same variation was not detected in the unaffected family members. Thus, this germline *RAD52* variation was most likely responsible for the molecular pathogenesis of GBC. Unfortunately, because the patient's half-brother died of GBC several years prior, at 56 years of age, and his father died of unknown cause, we could not determine whether they harbored the same variation (Fig. 1C).

In summary, we identified a novel *RAD52* mutation in a patient with GBC. Our results enlarged the mutation spectrum of the *RAD52* gene in patients with GBC. It also provides new insights into genetic counseling and targeted therapeutic strategies for patients with GBC.

## Acknowledgments

The authors thank the patient and his family, and all the personnel who participated in this study. We would like to thank Editage (www.editage.com) for English language editing.

## Author contributions

**Bioinformatics analysis**: Erhong Meng and Dongliang Wang.

**Conceptualization:** Xinzheng Dai.

**Formal analysis:** Erhong Meng, Dongliang Wang.

**Plotting figures**: Lei Yue and Jian Gu.

**RAD52 protein sequence alignment**: Xintong Wang.

**Software:** Erhong Meng.

**Visualization:** Lei Yue, Jian Gu, Xintong Wang.

**Writing – original draft:** Wenhu Zhao, Yongjiu Dai.

**Writing – review & editing:** Siyao Liu, Xinyin Han, Guojun Li.
